# Similar genetic profile in early and late stage urothelial tract cancer

**DOI:** 10.1007/s00432-024-05850-y

**Published:** 2024-07-08

**Authors:** Dag Rune Stormoen, Kristoffer Staal Rohrberg, Kent William Mouw, Katrine Ørum, Zoltan Szallasi, Maria Rossing, Frederik Otzen Bagger, Helle Pappot

**Affiliations:** 1 Department of Oncology, Copenhagen University Hospital Rigshospitalet, Blegdamsvej 9, Copenhagen, 5073 Denmark; 2 Department of Clinical Medicine, University of Copenhagen, Copenhagen, Denmark; 3 Department of Radiation Oncology, Dana Farber Cancer Institute, Boston, MA USA; 4 Harvard Medical School, Boston, MA USA; 5 Computational Health Informatics Program, Boston Children’s Hospital, Boston, MA USA; 6 Translational Cancer Genomics Group, Danish Cancer Society, Copenhagen, Denmark; 7 Department for Genomic Medicine, Copenhagen University Hospital Rigshospitalet, Copenhagen, Denmark

**Keywords:** Urothelial tract cancer, Genomic evolution, Targeted therapy, Molecular subtyping, Phase 1 trials

## Abstract

**Introduction:**

Urothelial tract cancer (UTC) ranks as the tenth most prevalent cancer and holds the seventh position in terms of mortality worldwide. Despite its prevalence and mortality ranking, there are still gaps in the knowledge of the mutational landscape in patients with advanced disease who have limited therapeutic options after multiple lines of prior treatment. This study compares the genomic and transcriptomic landscape, and targeted treatment options between metastatic UTC (mUTC) patients treated with multiple lines of therapy compared to newly diagnosed, untreated Muscle Invasive Bladder Cancer (MIBC).

**Methods:**

We compared genomic and clinical data from two cohorts: mUTC patients who received multiple lines of therapy and were referred to the Copenhagen Prospective Personalized Oncology (CoPPO) project at Rigshospitalet, University of Copenhagen. Data for MIBC UTC patients were acquired from the Cancer Genome Atlas Bladder Cancer (TCGA BLCA) cohort. Biopsies in CoPPO were performed at the time of enrollment. 523 highly important cancer-related genes (TrueSight Oncology-500 targeted sequencing panel) were used from both cohorts for comparative analysis. Analyses included RNA count data to compare predicted molecular subtypes in each cohort separately.

**Results:**

Patients from the CoPPO cohort had a lower median age at first-line treatment than the TCGA BLCA cohort, with no significant gender disparity. The predominant histology was urothelial cell carcinoma in both cohorts. Genomic analysis revealed no significant difference between the top mutated genes in the two cohorts, specifically looking into DNA damage repair genes. Molecular subtyping indicated a higher frequency of neuroendocrine differentiation in the CoPPO cohort. 13% of patients in the CoPPO cohort received targeted therapy based on genomic findings, and 16% received non-targeted treatment, totaling 29% receiving CoPPO treatment (9 patients). The remaining 71% received best supportive care. Kaplan-Meier analysis showed a non-significant survival benefit for the intervention group in the CoPPO cohort.

**Conclusion:**

When focusing on 523 highly relevant cancer genes, the mutational profile of mUTC patients who have undergone numerous treatment lines resembles that of newly diagnosed MIBC. These alterations can be targeted, indicating the potential advantage of early genomic testing for personalized treatment within clinical trials.

**Supplementary Information:**

The online version contains supplementary material available at 10.1007/s00432-024-05850-y.

## Introduction

Urothelial tract cancer (UTC) is the tenth most prevalent form of cancer worldwide and the thirteenth in terms of cancer-related mortality (Saginala et al. 2020; Sung et al. 2021). In early-stage, localized muscle-invasive urothelial tract cancer (MIBC), nearly half of the cases progress to metastatic disease [2,3] with a short survival due to disease progression (Westergren et al. 2019). UTC is more frequent in males than females (4:1) and is associated with smoking and certain environmental exposures (Freedman et al. 2011; Safiri et al. 2021).

Until recently, first-line therapy for mUTC includes cisplatin-based chemotherapy for eligible patients, with carboplatin-based regimens as an alternative for those who cannot tolerate cisplatin (Witjes et al. 2021; Cathomas et al. 2022). These regimens slow disease progression, reduce symptoms, and prolong survival. Before chemotherapy, median overall survival ranged from 3 to 6 months (Loehrer et al. 1992); with platinum treatment, this was extended to 12–16 months(Loehrer et al. 1992; Von der Maase et al. 2000; Bellmunt et al. 2012), maintenance avelumab increased survival to 23 months (Powles et al. 2020, 2023), and recent data shows favorable outcomes with the first-line combination of enfortumab-vedotin and pembrolizumab, increasing median survival to 31.5 months (Hoimes et al. 2023; O’Donnell et al. 2023; Powles et al. 2024). The low levels of evidence for targeted therapies in large repositories like OncoKB(Chakravarty et al. 2017) indicate a knowledge gap in precision medicine for metastatic UTC (mUTC), underscoring the need for more clinical research to validate additional targeted treatments.

It has been suggested that systemic treatments exert selective pressure that can alter the genomic profile of advanced and mUTC (Venkatesan et al. 2017), highlighting the importance of identifying lethal tumor cell clones and understanding genomic diversity for improving patient outcomes. This knowledge is crucial for customizing treatments to target genetic drivers (Meeks et al. 2020). Beyond FGFR mutational status for targeted therapy (Loriot et al. 2019), little clinical evidence exists for targeting mutations in UTC despite its high mutational burden (Robertson et al. 2017; Kamoun et al. 2020; Damrauer et al. 2022). Several trials have examined the effect of exploiting the synthetic lethality of homologous repair deficiency (HR deficiency) with poly ADP-ribose polymerase inhibitors (PARPis), as single-agent or in combination with immune checkpoint inhibitors (ICIs) (Fulton et al. 2020; Grivas et al. 2021; Powles et al. 2021a; Crabb et al. 2023; Vignani et al. 2023; Gamba et al. 2023), with varying results, and further trials will determine the role of PARPi for mUTC. In mUTC, a range of therapeutic targets encompasses genes involved in several key mechanisms, including signal transduction and kinase activity, DNA damage response and repair, chromatin modification and gene expression regulation, cell cycle regulation, metabolic pathways, and tumor growth and angiogenesis (genes and pathways summarized in Table S1) (Robertson et al. 2017).

The genomic landscape of metastatic urothelial tract cancer is characterized by genetic heterogeneity (Lavallee et al. 2021), complicating treatment strategies. This heterogeneity is not solely intertumoral but also intratumorally manifested at genomic, transcriptional, and cellular levels, leading to variations in therapeutic effectiveness (Schulz et al. 2020; Meeks et al. 2020).

The genomic variability also encompasses diverse histology and morphology, including urothelial (transitional cell), adenocarcinoma, micropapillary, squamous, and small-cell carcinoma types, where urothelial carcinoma is the dominant histological type, accounting for 90–95% of cases (Humphrey et al. 2016). Although not currently clinically implemented, molecular subtyping of UTC has offered further means to describe tumor characteristics through bulk RNA analysis (Kamoun et al. 2020) and immunohistochemistry (Sjödahl 2018; Sjödahl et al. 2022; Goutas et al. 2023). The clinical effect of molecular subtyping and which classifier method to rely on remains to be fully understood (Morera et al. 2020).

mUTC exhibits a complex and dynamic genomic profile with high somatic mutation rates, paralleling melanoma and lung carcinoma (Robertson et al. 2017). The comprehensive analysis by The Cancer Genome Atlas (TCGA) Research Network has enhanced the understanding of molecular alterations in urothelial carcinomas, providing comprehensive data from the treatment-naïve MIBC setting (Robertson et al. 2017).

In the present study, we compare genomic alterations in mUTC samples from patients who received multiple lines of treatment with untreated MIBC patients (TCGA cohort) and review potential targetable treatment options, focusing on 523 cancer-relevant genes.

## Methods and material

### Data acquisition

#### The copenhagen prospective personalized oncology (CoPPO) cohort

Patients who have exhausted standard treatment options or are moving toward exhaustion can, in Denmark, be referred to the Phase 1 unit at Rigshospitalet for genomic testing and screening for Phase 1 trials and CoPPO inclusion. Patient recruitment was conducted from May 2013 to December 2022 (ongoing), and patients with urothelial tract carcinoma referred to the phase I unit were considered for enrollment. The inclusion criteria were: patients had exhausted all treatment options, a life expectancy of at least three months, normal organ function, presence of measurable disease according to RECIST 1.1 criteria, an Eastern Cooperative Oncology Group (ECOG) performance status (PS) of 0 or 1, age of 18 years or above, and having lesions amenable to biopsy. Data collection is described in detail in a previously published paper (Tuxen et al. 2019).

Biopsy samples were predominantly obtained from metastatic sites under local anesthesia. The methods of biopsy included ultrasound guided core-needle biopsies from same lesion using an 18-gauge needle or surgical resection. Three samples were collected from each lesion. Two of these samples were preserved in RNA later (Life Technologies) for subsequent RNA expression and DNA gene mutation analyses. The third sample underwent formalin fixation and paraffin embedding (FFPE) for histopathological examination. Additionally, a blood sample (7 mL, EDTA) was drawn to analyze germline variants. If a biopsy, for logistical or technical reasons, could not be performed, circulating tumor DNA (ctDNA) was extracted from the blood sample (Tuxen et al. 2019).

#### DNA and RNA extraction and sequencing platforms

DNA and RNA were extracted from tumor samples preserved in RNA later using the AllPrep DNA/RNA/protein Extraction Kit (Qiagen), according to the manufacturer’s instructions. The Tecan automated liquid handling station was employed for DNA extraction from whole blood samples. Circulating DNA was extracted from 2 to 4 ml plasma using the QIAsymphony Circulating DNA Kit (Qiagen, Hilden, Germany) according to the manufacturer’s instructions with an elution volume of 60 μl. Collection and analysis of peripheral blood for germline and ctDNA is described in detail in (Ahlborn et al. 2017, 2018). Germline DNA was sequenced on the Illumina NextSeq platform to a minimum average coverage of > 50x as described in (Ahlborn et al. 2018).

Whole Genome Sequencing (WGS) was conducted with targeted sequencing depth of 90x, a minimum average sequencing depth of 60x, and a variant allele frequency (VAF) threshold of at least 10%. For circulating tumor DNA (ctDNA), the Illumina TruSight Oncology (TSO500) panel or Whole Exome Sequencing (WES) was used, requiring an average sequencing depth of 600x and a VAF threshold of 2%. For FFPE samples sequenced, we applied the TSO500 panel; an average sequencing depth of at least 300x and a VAF threshold of at least 5% were required. Across all sequencing methods, a baseline of a minimum of 10 reads was required. The choice of analysis platform (WGS, WES, TSO500) depended on when the patient was referred and which tissue was available for analysis (i.e., blood, FFPE, or fresh frozen tissue).

For the preparation of WES, the SureSelect Clinical Research Exome (Agilent) kit was used for both tumor and germline DNA, including a fragmentation to 300 bp with Covaris S2 (Agilent) and adaptor ligation using the KAPA HTP Library Preparation Kit (Roche). WES and RNA sequencing libraries were sequenced using paired-end sequencing on the Illumina NextSeq500 or HiSeq2500 platforms. Sequence alignment against the human reference genome (hg38/GRCh38) was performed using Sentieon-genomics (version 202,112 ) BWA-mem counterpart. Somatic mutations were reported through tumor/normal joint analysis, functionally subtracting germline variants from tumor variants using Strelka2 (version 2.7). RNA count data were aquired from raw sequencing reads using STAR (version 2.7.10a) and quantified using HTSeq and normalized using the median of ratios, DESeq2 v.1.42.0 R-package for subsequent molecular subtyping analysis.

#### TCGA BLCA cohort

Genomic, expression, and clinical data on 412 muscle-invasive bladder cancer (MIBC) patients were downloaded using the “TCGA biolinks” package [36] in R. All data was aligned to the GRcH38 reference genome. In the analysis of The Cancer Genome Atlas (TCGA) DNA data, reads were filtered to include only those with a minimum of 10 reads and a VAF of 10%. This was followed by sequencing quality filtration and the exclusion of common variants at a frequency greater than 1% in the 1000 Genomes, ExAC, and NHLBI ESP databases. RNA count data were normalized using the median of ratios, DESeq2 v.1.42.0 R-package for molecular subtype analysis.

#### Variant annotation and filtering

We used the variant and minimum allele frequencies specified in Figure S1; we applied this filtering for the TCGA BLCA and CoPPO cohorts. For annotation of variants, we used variant effect predictor (VEP, release 111, assembly reference genome GRCh38.p14)(McLaren et al. 2016) and vcf2maf (https://github.com/mskcc/vcf2maf). OncoKB annotation was performed using the OnkoKB MAF annotator (https://github.com/oncokb/oncokb-annotator), using the API service offered by OncoKB (Chakravarty et al. 2017; Suehnholz et al. 2023). As targeted sequencing (TSO500) was used on a portion of the CoPPO cohort, both cohorts were filtered to exclude genes not covered by the panel (Appendix 1.1). To compare relevant DDR genes of interest between the cohorts, we filtered for a subset of DDR genes (Appendix 1.2).

#### Molecular subtype analysis

The molecular subtype for the samples with available RNA sequencing data was conducted using normalized RNA counts, with the R script described in(Robertson et al. 2017) available at (“https://github.com/cit-bioinfo/consensusMIBC” and “https://github.com/cit-bioinfo/BLCAsubtyping”). We classified the samples for Cartes d’Identité des Tumeurs (CIT)-Curie(Rebouissou et al. 2014), University of North Carolina (UNC) (Damrauer et al. 2014), MD Anderson Cancer Center (MDA)(Choi et al. 2014), Lund (Marzouka et al. 2018), TCGA (Robertson et al. 2017), and Consensus type (Kamoun et al. 2020).

#### Statistical analysis

We used the Fisher exact test to assess the difference between distinct variables between the CoPPO and TCGA BLCA cohort; the Welch T-test was applied for continuous variables. Were stated, we corrected for multiple testing using the Benjamini-Hochberg (BH) procedure. Survival duration for the CoPPO cohort was defined as the time from initiating first-line therapy for metastatic disease and, secondly, from the time of inclusion in the CoPPO trial until death or censoring. Survival duration for the TCGA cohort was defined as the time from pathological diagnosis until death or censoring. Survival was assessed using Kaplan-Meyer, with a log-rank test to discern the statistical differences between the selected strata. All analyses were performed in R version 4.3.1 (R Core Team (2020).), and maftools v.2.18.0(Mayakonda et al. 2018) were used to visualize genomic data.

### Ethical considerations

All patients in the CoPPO cohort gave written informed consent to participate in the trial. The study was approved by the Danish Data Protection Agency (j.no.: 2012-58-004) and the Regional and National Ethics Committees (file numbers 1,300,530 and H-16,046,103, respectively). We have adhered to the declaration of Helsinki (2013). Data from the TCGA cohort is publicly available from the GDC repository (https://gdc.cancer.gov/publication-tag/tcga-blca).

## Results

After filtration, 30 (of 31) samples from the CoPPO cohort had genomic data available for further analysis, and all 31 patients were included in the survival analysis. From the TCGA cohort, 401 (of 412) samples were included after filtering. The patients in the CoPPO cohort had a significantly lower median age at first-line treatment than in the TCGA BLCA cohort. The gender distribution between the cohorts shows no significant difference (Table 1). Performance Status (PS) was only available for 24.5% of patients in the TCGA cohort, making comparative analysis obsolete; PS for the CoPPO Cohort was 0–1 for all patients at the time of biopsy (inclusion criteria). In the CoPPO Cohort, all patients had previously received platinum treatment as first-line therapy, with 90% receiving a cisplatinum-containing regimen. Twenty-nine (94%) patients received 2nd line treatment, with ICI constituting 46% of 2nd line treatments. Third-line treatment was administered to twelve patients (39% of 31 patients); vinflunine was given to six patients (19% of 31 patients) (Table 1). Nine patients received treatment in the CoPPO cohort, six as third-line treatment, and three as 4th line treatment (Table S2S). Of the nine patients in the CoPPO cohort, four received targeted therapy and five non-targeted therapy. The patients not qualifying for treatment in the CoPPO cohort received best supportive care (BSC).

**Table 1:** Clinical characteristics of patients. (p-value: ns: “>0.05”, Welch T-test for age difference, Fisher exact test for discrete variables)

Clinical characteristics	CoPPO cohort (*n* = 31)	TCGA cohort (*n* = 401)	*p*-value
Age (at first-line treatment), median (range)	63 (48–76) years	69 (34–90) years	0.0011
Age at CoPPO trial	65 (48–77) years		
Time from first-line treatment to CoPPO, median (range)	13 (2.4–60.4) months		
Female n (%)	10 (32%)	103 (26%)	ns
Male n (%)	21 (68%)	298 (75%)	ns
***Histology***:			
Urothelial carcinoma, n (%)	25 (81%)	333 (83%)	ns (0.804)
Small cell endocrine, n (%)	2 (6%)	0 (0%)	0.005
Squamous cell carcinoma	4 (13%)	1 (0.2%)	0.0001
Carcinoma, not otherwise specified	0 (0%)	1 (0.2%)	ns
Papillary adenocarcinoma	0 (0%)	1 (0.2%)	ns
Papillary urothelial cell carcinoma	0 (0%)	65 (16.2%)	0.008
***Previous treatment***:			
**1st line**	**31 patients (100%)**		
MVAC	2		
Carboplatin/gemcitabine	2 (6%)		
Carboplatin/gemcitabine/topo	1 (3%)		
Cisplatin/gemcitabine	26 (84%)		
**2nd line**	**29 patients (94%)**		
Atezolizumab	3 (10%)		
Avelumab maintenance	1 (3%)		
Pembrolizumab	10 (32%)		
Taxol/xeloda	1 (3%)		
Vinflunin	8 (26%)		
Vinflunin + Pemetrexed	1 (3%)		
carbo/gemc	2 (6.5%)		
cis/gem reind	3 (10%)		
No 2nd line treatment	2 (6.5%)		
**3rd line**	**12 patients (39%)**		
Carbo/gemc (reinduction)	2 (7%)		
Enfortumab-vedotin (protokol)	1 (3%)		
Olaparib	1 (3%)		
Pembrolizumab	2 (7%)		
Vinflunin	6 (19%)		
No standard third-line treatment	19 (61%)		
**CoPPO treatment**	**9 patients. (29%)**		

The predominant histological diagnosis in both cohorts was urothelial cell carcinoma. The TCGA cohort has a notable overrepresentation of papillary urothelial cell carcinoma. Conversely, the CoPPO cohort demonstrates a higher incidence of squamous and small-cell endocrine carcinoma, although the sample sizes for these subtypes are small (Table 1).

**Fig. 1:**
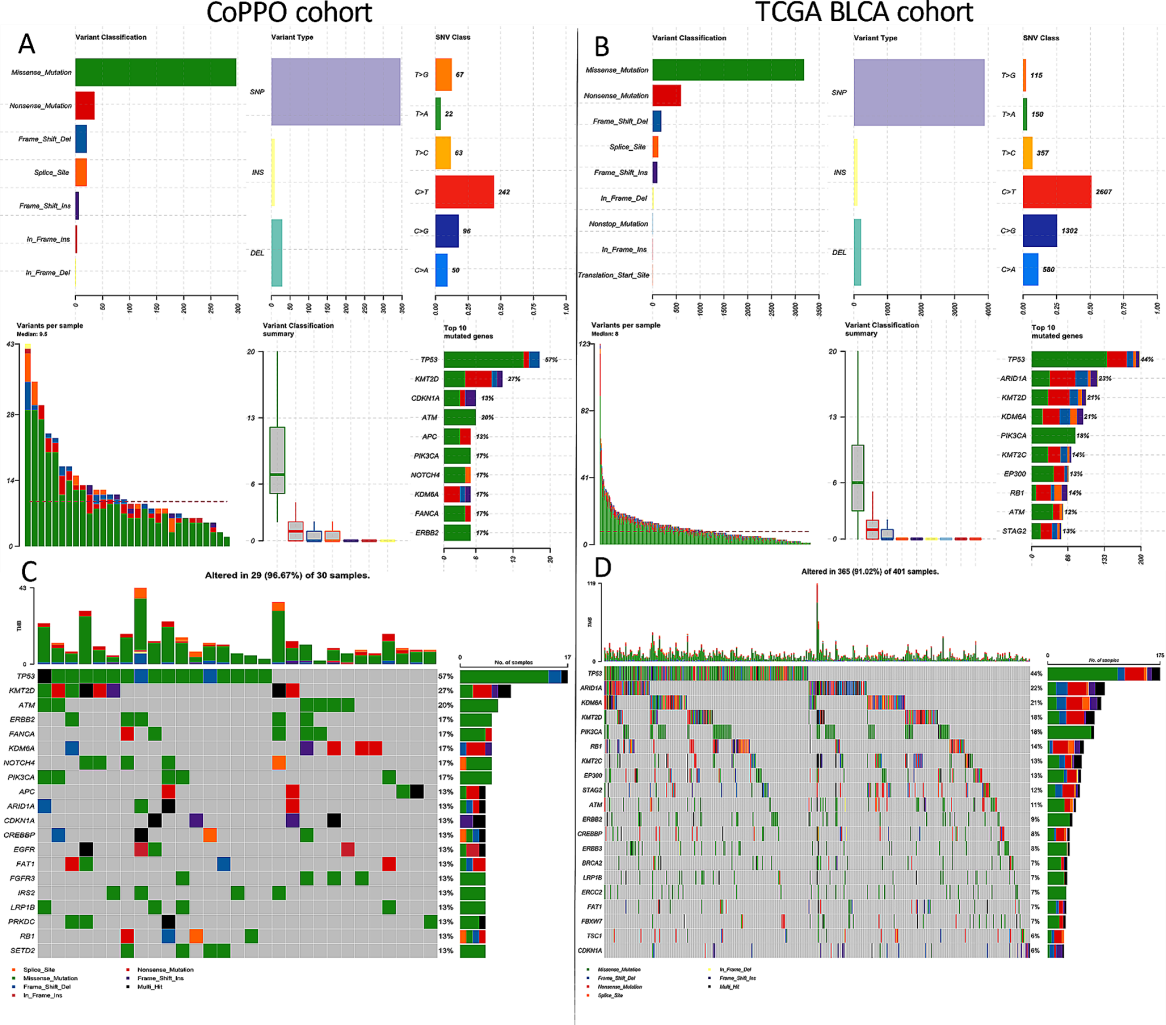
Summary plots for **A**: CoPPO and **B**: TCGA. Oncoplots for top 20 mutated genes in **C**: CoPPO cohort, **D**: TCGA cohort

**Fig. 2:**
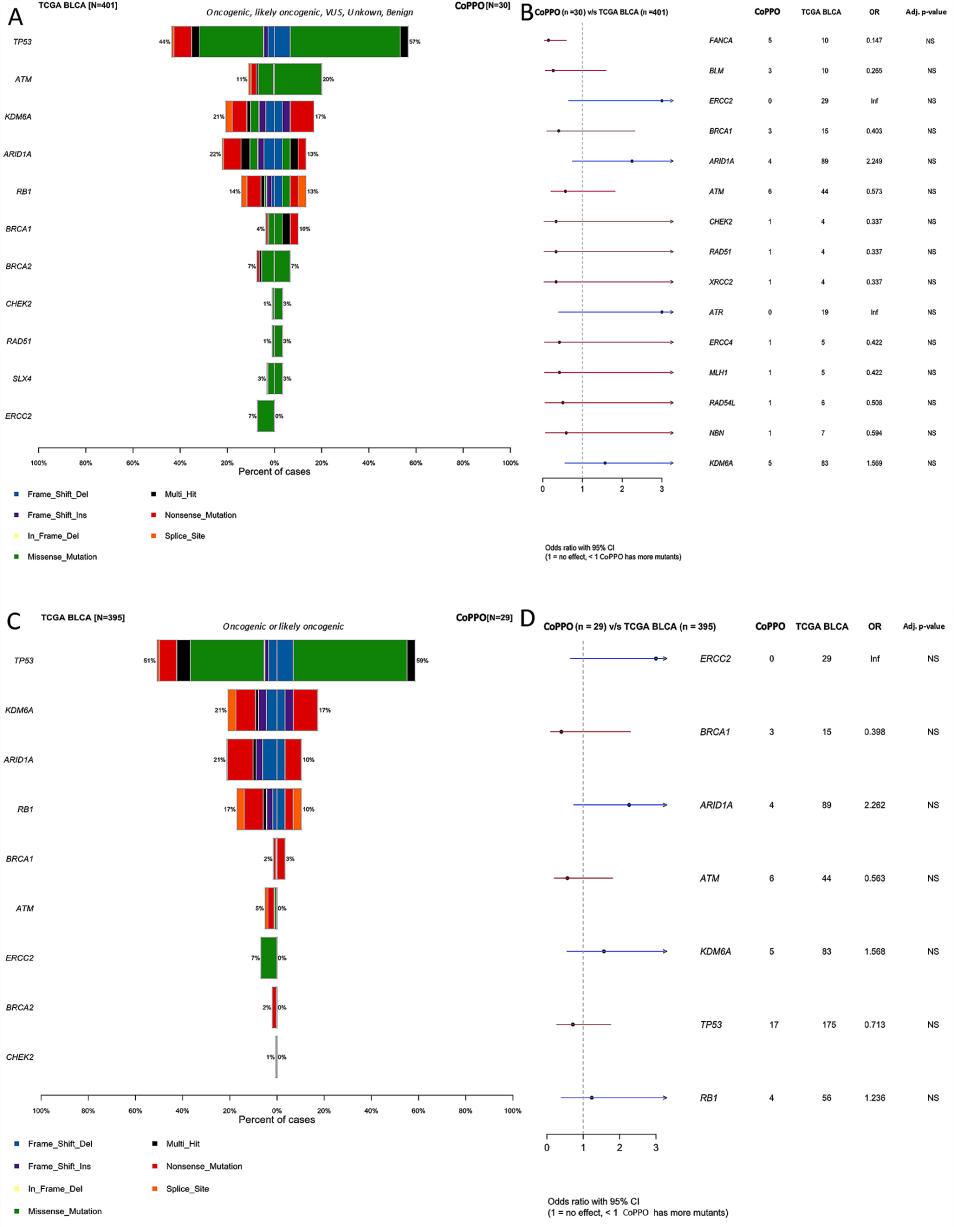
**A**: Cobarplot of top mutated DDR-relevant genes in TCGA (left) and CoPPO cohort (right), including variants of unknown significance (VUS), benign, likely oncogenic and oncogenic variants. **B**: Forest plot showing the difference, adjusted p-values not shown, all non-significant. **C**: Cobarplot of likely oncogenic and oncogenic variants from TCGA (left) and COPPO (right). **D**: Forest plot of statistical difference between the groups. NS = non-significant. Variant annotation according to pathogenicity by the OncoKB database (Chakravarty et al. 2017; Suehnholz et al. 2023)

Of the 523 genes in the TSO500 targeted sequencing panel of highly relevant cancer genes, we found no difference in top mutated genes between CoPPO and the TCGA cohort or any significant difference between mutations in DDR genes (Figs. 1 and 2). Briefly, the most frequent mutations in the CoPPO cohort (including variants of unknown significance (VUS)) were TP53 (57%), KMT2D (27%), ATM (20%), ERBB2 (17%), and FANCA (17%). For the TCGA Cohort, the most frequent mutations were TP53 (44%), ARID1A (22%), KDM6A (21%), KMT2D (18%), and PIK3CA (18%) (Fig. 1). After filtering for oncogenic or likely oncogenic variants, the top mutations in the CoPPO cohort were TP53 (59%), KMT2D (21%), KDM6A (17%), PIK3CA (17%), and FGFR3 (17%). For the TCGA cohort, TP53 (51%), ARID1A (21%), KDM6A (21%), KMT2D (19%), and PIK3CA (18%) (Figure S2). The most frequent type of mutation in both cohorts was missense mutations, and the most frequent single nucleotide shift was C > T (Fig. 1A and B). Filtering for DDR genes and oncogenic/likely oncogenic variants, no statistical difference emerges, notably though no ERCC2 or ATM mutations are present in the CoPPO cohort (Fig. 2C and D). Top VAFs before and after filtering for oncogenic or likely oncogenic variants are shown in Figure S3. Notably, we did not find any significant difference in the frequency of mutations among the 523 genes between the two cohorts.

### Molecular subtype

Nineteen patients with genomic results had accompanying RNA analysis performed in the CoPPO cohort. Neuroendocrine differentiation frequency was higher in the CoPPO cohort than in the TCGA BLCA cohort (Table 2). No difference between luminal or basal subtypes was observed between TCGA BLCA and CoPPO (Table S3).

**Table 2:** Showing significant and borderline significant differences between molecular subtypes in the CoPPO cohort (*n* = 19) and the TCGA cohort (*n* = 431 samples). * full overview of molecular subtypes depicted in table S3

Classifier	Subtype	Count TCGA	Count CoPPO	Percentage TCGA	Percentage CoPPO	*p*	*p*.adj	*p*.adj.signif
CIT	MC3	17	4	3.9	21.1	0.01	0.07	ns
CIT	MC6	3	3	0.7	15.8	0.00	0.01	*
Lund	Sc/NE-like	15	4	3.5	21.1	0.01	0.06	ns
TCGA	Neuronal	19	6	4.4	31.6	0.0001	0.01	**
Consensus	NE-like	6	4	1.4	21.1	0.0001	0.01	**

### Survival outcomes

**Fig. 3:**
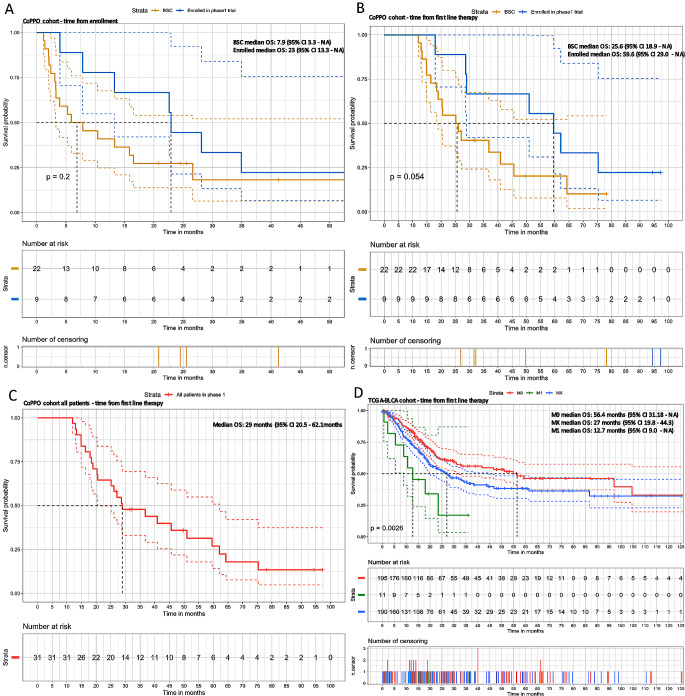
Survival curves of CoPPO cohort. **A**: From the time of enrollment in CoPPO. **B**: From the time of baseline first-line treatment. **C**: Survival of all patients referred to the CoPPO unit from first-line treatment. **D**: Survival of TCGA-BLCA cohort stratified by metastasis at baseline first-line treatment. M0: No metastatic disease at baseline, M1: Metastatic at baseline, MX: Metastasis cannot be measured

The Kaplan-Meier survival analysis (Fig. 3) illustrates the observed duration from enrollment in the phase 1 trial (Fig. 3A) and from baseline first-line treatment (Fig. 3B). The survival probabilities for the cohort receiving the intervention remain higher than for patients not receiving CoPPO treatment throughout the study period, although small patient numbers should be noted. Divergence occurs early and persists, although not statistically significant (*p* = 0.2). Survival for CoPPO patients is combined in Fig. 3C, showing a median survival of 29 months from the start of first-line therapy. For the TCGA cohort, which comprises primarily patients with non-metastatic (M0) or unmeasurable metastatic disease (MX), the 11 patients with known metastatic disease have shorter survival than MX and M0 (*p* = 0.0026) (Fig. 3D) and markedly shorter survival than the metastatic patients included in the CoPPO cohort (not statistically compared).

### Targetable mutations

**Fig. 4:**
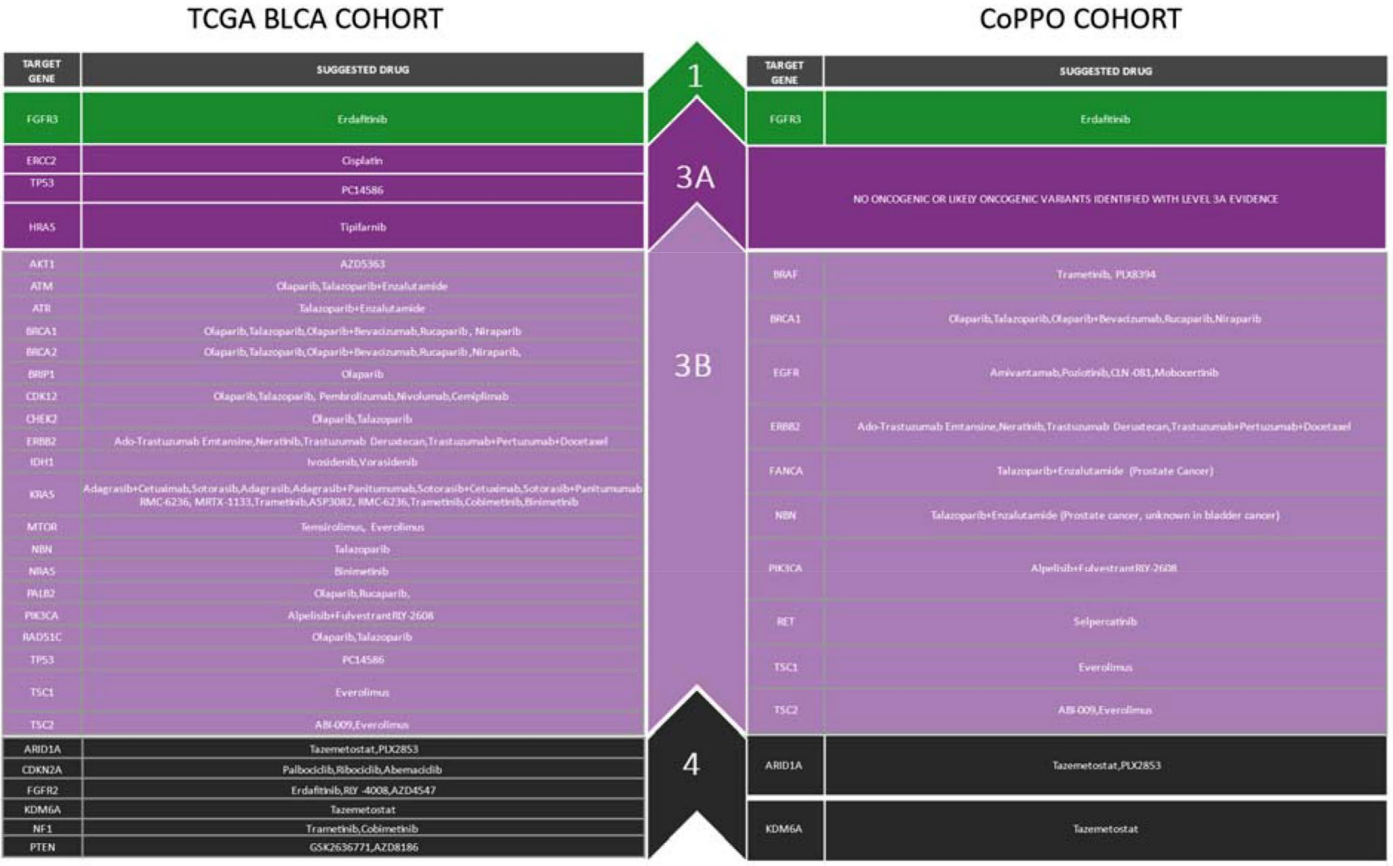
Treatment options, according to OncoKB, for TCGA and CoPPO are only oncogenic and likely oncogenic. All variants and references are listed in Appendix 2. Level 1: FDA-recognized biomarker predictive of response to an FDA-approved drug in this indication. Level 2: Standard care biomarker recommended by NCCN or other professional guidelines predictive of response to an FDA-approved drug in this indication. Level 3 **A**: Compelling clinical evidence supports the biomarker as predictive of a drug’s response in this indication. Level 3 **B**: Standard care or investigational biomarker predictive of response to an FDA-approved or investigational drug in another indication. Level 4: Compelling biological evidence supports the biomarker as predictive of drug response (Suehnholz et al. 2023)

Figure 4 compiles the oncogenic or likely oncogenic variants where drugs targeting these genomic alterations are available for the TCGA BLCA and CoPPO cohorts. These include kinase inhibitors, DNA-damage response modulators, CDK inhibitors, mTOR inhibitors, and IDH inhibitors, as detailed in Fig. 4 and Tables S1 and S4. Four patients in our cohort received targeted therapy (Table S2) directed at mutations in ERBB2 or FGFR3, and five patients received non-targeted treatment; no survival difference between targeted and non-targeted therapy was observed (Figure S4), although small patient numbers limit this interpretation.

## Discussion

The present study explores the mutational landscape of mUTC, focusing on 523 highly relevant cancer genes, comparing patients who received multiple lines of treatment with untreated patients, and examining possible targetable alterations.

Using the OncoKB repository, we identified targeted treatment options for personalized cancer therapy across genetic mutations (Chakravarty et al. 2017). Only Erdafitinib, targeting FGFR3 mutations, received OncoKB Level 1 (FDA-approved indication). For BRCA mutations, Level 3 evidence supports PARP inhibitors (Olaparib, Rucaparib, Niraparib) in cell cycle and DNA repair pathways. Trametinib is suggested for BRAF mutations under the MAPK pathway, and HER2-directed therapies, like Ado-Trastuzumab Emtansine and neratinib for ERBB2 mutations, are indicated by Level 3 evidence (Fig. 4).

Genomic alterations, influenced by factors such as APOBEC enzymes, DNA-repair anomalies, and therapeutic interventions, can impact the response to treatments in urothelial tract cancer by promoting tumor evolution. APOBEC enzymes, while vital in immune defense and RNA editing, contribute to cancer development by inducing mutations (Robertson et al. 2017). This leads to genomic instability, potentially activating or deactivating essential cancer-related genes. APOBEC enzymes increase the mutational burden in UTC by converting cytosine to uracil in DNA, leading to C-to-T mutations, the most frequent single nucleotide variation (SNV) in UTC (Robertson et al. 2017). These alterations can affect oncogenes and tumor suppressor genes, playing a role in the initiation and progression of the disease. Interestingly, we did not find a difference in mutation rate between the two cohorts, among the 523 examined cancer related genes.

The absence of ERCC2 mutations in the CoPPO cohort is noteworthy. One plausible explanation for this observation is the potential for a better long-term complete response to chemotherapy in patients with ERCC2 mutations. This might lead to their exclusion from this cohort that has received multiple lines of therapy (Van Allen et al. 2014; Li et al. 2019). This hypothesis aligns with the understanding that genetic alterations can influence treatment efficacy (Van Allen et al. 2014; Li et al. 2019). Furthermore, the finding that none of the 20% of ATM mutations in the CoPPO cohort was classified as oncogenic or likely oncogenic contrasts with the TCGA cohort, where the reduction was less marked. This discrepancy might indicate a selective elimination of pathogenic ATM and ERCC2 mutations due to previous treatments, hinting at the evolutionary pressure exerted by therapeutic interventions on the genomic profile of mUTC, as patients with these mutations are thought to have a better response to platinum treatment (Birkbak et al. 2012; Van Allen et al. 2014; Peng et al. 2014; Liu et al. 2016; Wang et al. 2016; Hu et al. 2018; Li et al. 2019). However, this occurrence might also be influenced by the limited sample size of the CoPPO cohort, which could affect the detection and representation of genetic mutations. The smaller cohort size may not adequately capture the genetic diversity, potentially leading to a skewed understanding of mutation prevalence. Additionally, ATM spans over approximately 150 kilobases and comprises 66 exons, where most mutations have not been manually curated or examined, which can explain the absence of oncogenic variants in the CoPPO cohort (Waskiewicz et al. 2021). The large size of the ATM gene and its complex exon-intron structure pose challenges for identifying oncogenic variants, given the potential for mutations and variations across the sizeable genomic region.

RNA-based molecular subtyping partially corroborates the histological findings of a higher prevalence of small-cell neuroendocrine features in the CoPPO cohort. Detecting additional subtypes with neuroendocrine features through molecular analysis underscores the genomic heterogeneity and evolutionary complexity of mUTC. This diversity has implications for treatment strategies, as it highlights the need for personalized approaches tailored to the molecular characteristics of each tumor. However, the clinical implication of molecular subtyping is under debate (Patschan et al. 2015; Mitra 2016; Sjödahl et al. 2017, 2019, 2022; Eich et al. 2017; Peeker 2018; Bernardo et al. 2019; Morera et al. 2020).

The CoPPO cohort, comprising 31 patients over eight years, is characterized by its highly selective nature. This selection was based on criteria that included patients in good physical condition compared to the typical first-line mUTC treatment population. The implication of such selection is twofold. Firstly, these patients’ inherently better health likely contributes to their extended survival rates, a factor independent of the specific treatments administered in CoPPO (Fig. 3C). Secondly, this disparity in baseline health status complicates direct comparisons between the CoPPO cohort, which comprises metastatic cases, and the TCGA cohort, which primarily includes adjuvant cases. However, comparing the mutational landscape is valuable as this informs us about the cancer’s genetic evolution.

Furthermore, the intensive treatment regimen for patients enrolled in trials in the CoPPO cohort (nine patients), which involves frequent visits to the clinic – often several times per week – provides these patients with additional supportive care. This regular and close medical attention could play a role in the observed prolongation of survival (Fig. 3A and B). Such a high level of care, encompassing medical treatment and supportive services, is not as readily available or consistent in standard supportive care settings. Therefore, one should consider the impact of this extra care when evaluating the CoPPO cohort’s survival outcomes. However, trial inclusion has not impacted survival within other disease areas (Merkhofer et al. 2021).

Considering the presented findings, this study elucidates prospective pathways for research and clinical practice. Primarily, genomic testing in mUTC is underutilized for detecting mutations amenable to targeted therapy. Our findings suggest that early integration of genomic profiling and targeted treatment strategies in the mUTC disease course may give more treatment options. Most patients’ physical health declines rapidly after two to three lines of conventional treatment, which is why early integration is vital. This decline in performance status often prevents further therapeutic interventions, even in cases where targetable lesions are present. Therefore, the early adoption of genomic profiling is crucial in identifying and exploiting therapeutic targets before the patient’s condition worsens and precludes such interventions.

Secondly, exploring novel therapeutic modalities, particularly for patients exhibiting specific mutations or molecular subtypes, is paramount. We highlight several pharmacological agents targeting common mutations in mUTC. As illustrated in Fig. 4 and elaborated upon in Appendix 2, these agents have yet to be extensively tested in mUTC. A comprehensive clinical trial framework could address this, akin to the BISCAY trial (Powles et al. 2021b) but encompassing a more comprehensive array of targets and therapeutic agents. This approach aligns with the growing potential of targeted therapies in mUTC, which are currently non-existent in standard treatment regimens.

We acknowledge certain limitations that warrant consideration. A primary limitation is the sample size of the cohorts, as it limits the statistical power, particularly in survival analysis. The small cohort size of the CoPPO cohort, is important when examining less common genetic mutations or subtypes, which might be underrepresented or not captured in a limited sample. Additionally, stringent filtering criteria for genomic data have been employed. While this approach was adopted to minimize the inclusion of artifacts or irrelevant variants, it will exclude potentially pathogenic variants. Several different laboratory protocols were used, with different signal and noise profiles. Notably, ctDNA requires expert interpretation, just like the ends of exons from WES, which are known to have low quality. Allthough, multiple studies have shown comparable results between ctDNA and tissue genomics given that data are appropriately managed(Vandekerkhove et al. 2019; Carroll et al. 2019) in mUTC, and both protocols are applied routinely in clinical practice. The RNA-seq data that was used for subtype prediction was either poly-A capture (TCGA) or total RNA (CoPPO), in addition to the inter-lab RNA quantification batch effects that are to be expected. This difference could thus potentially affect subtype prediction accuracy or comparability, depending on the robustness of the tested tools.

## Conclusion

We found no statistical difference in the mutational landscape between patients screened for CoPPO trials and the TCGA BLCA cohort. Numerous targetable genomic alterations exist for mUTC patients, and drugs targeting these lesions are available. Initiating genomic testing early in the disease trajectory can provide more personalized treatment options for mUTC patients in a clinical trial setting.

### Electronic supplementary material

Below is the link to the electronic supplementary material.

Supplementary Material 1

Supplementary Material 2

Supplementary Material 3

Supplementary Material 4

## Data Availability

Data is provided within the manuscript or supplementary information files. Raw data is not allowed per Danish law to be shared, but specific data on genes can be shared, by mail to corresponding author.
