# Supplementary material for: Similar genetic profile in early and late stage urothelial tract cancer
Source: J Cancer Res Clin Oncol. 2024 Jul 8;150(7):339. doi: 10.1007/s00432-024-05850-y (PMC11230994; doi:10.1007/s00432-024-05850-y)
Supplement: Supplementary file 4 — Supplementary Material 4 [file 432_2024_5850_MOESM4_ESM.docx]

Supplementary material


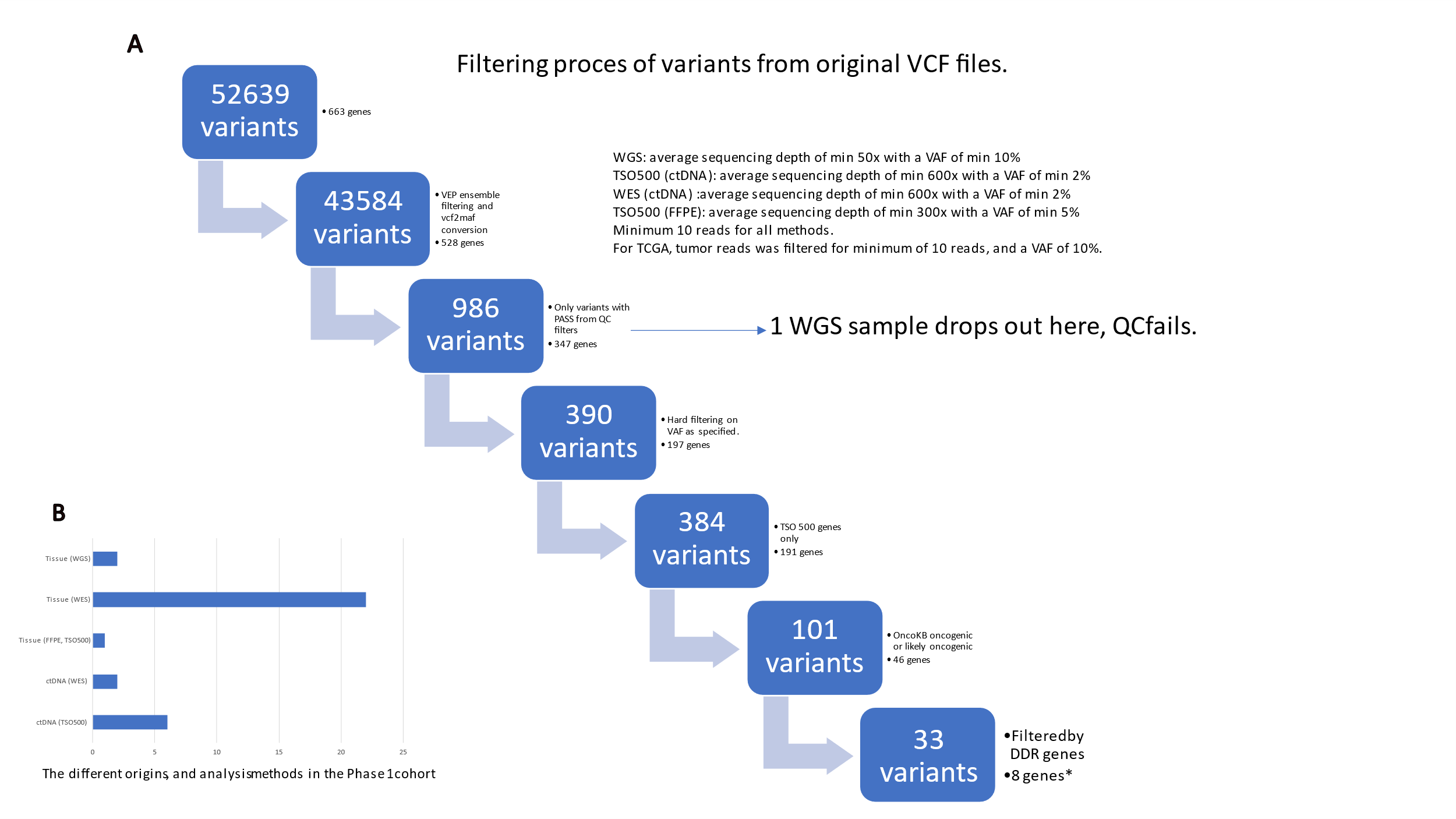


Figure S1: A: Filtering process of Phase 1 cohort. B: Analysis method of samples. A total of 31 samples, where 1 sample fails QC checks, resulting in 30 samples with genomic analysis from the CoPPO cohort.


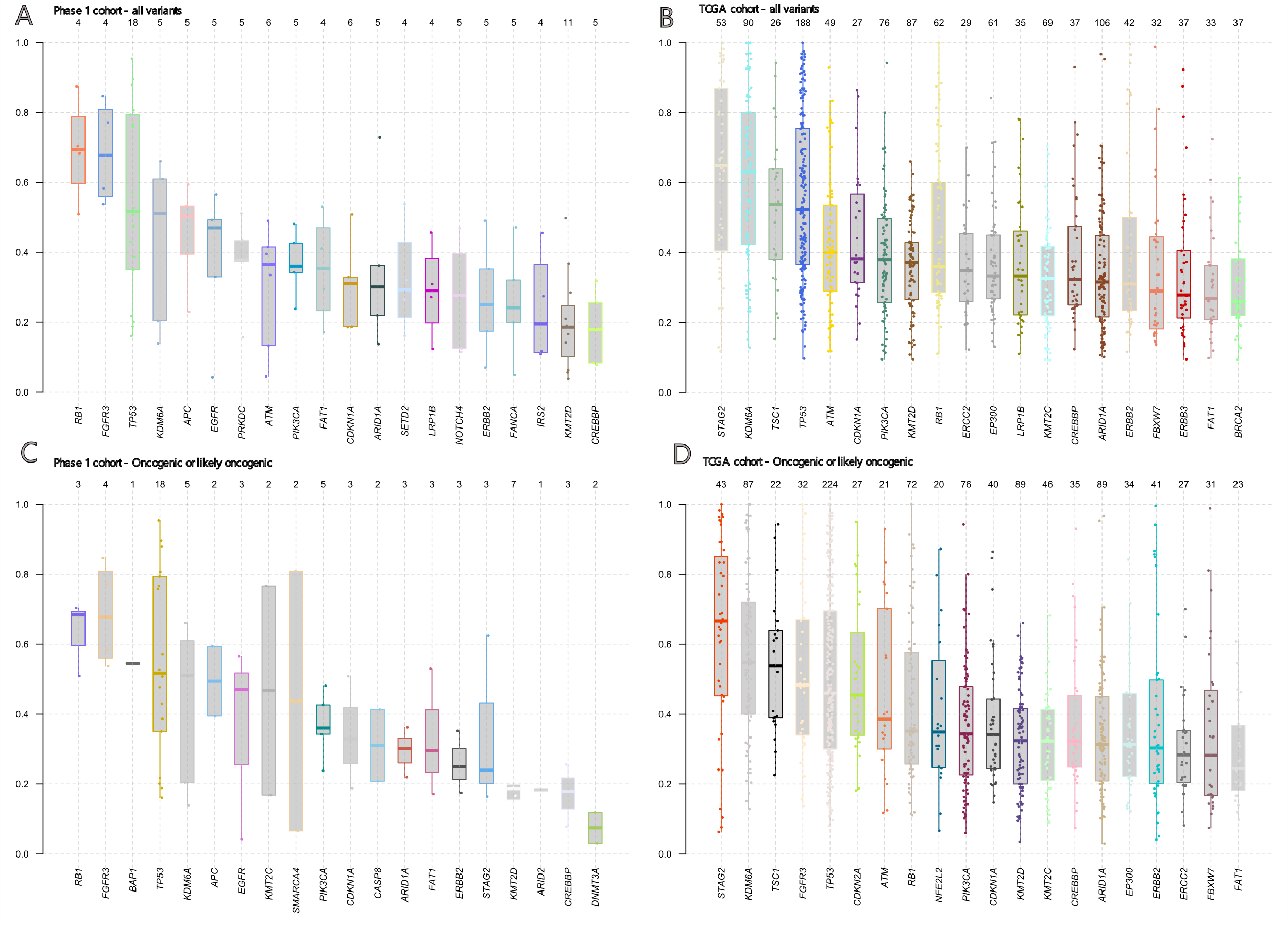


Figure S3: Showing top 20 variant allele frequencies after hard filtering and filtering for TSO-500 genes of A: Phase 1 cohort, all variants. B: TCGA – all variants, C: Phase 1 cohort oncogenic or likely oncogenic variants, D: TCGA-cohort oncogenic or likely oncogenic variants. Pathogenicity of variants classified according to OncoKB.


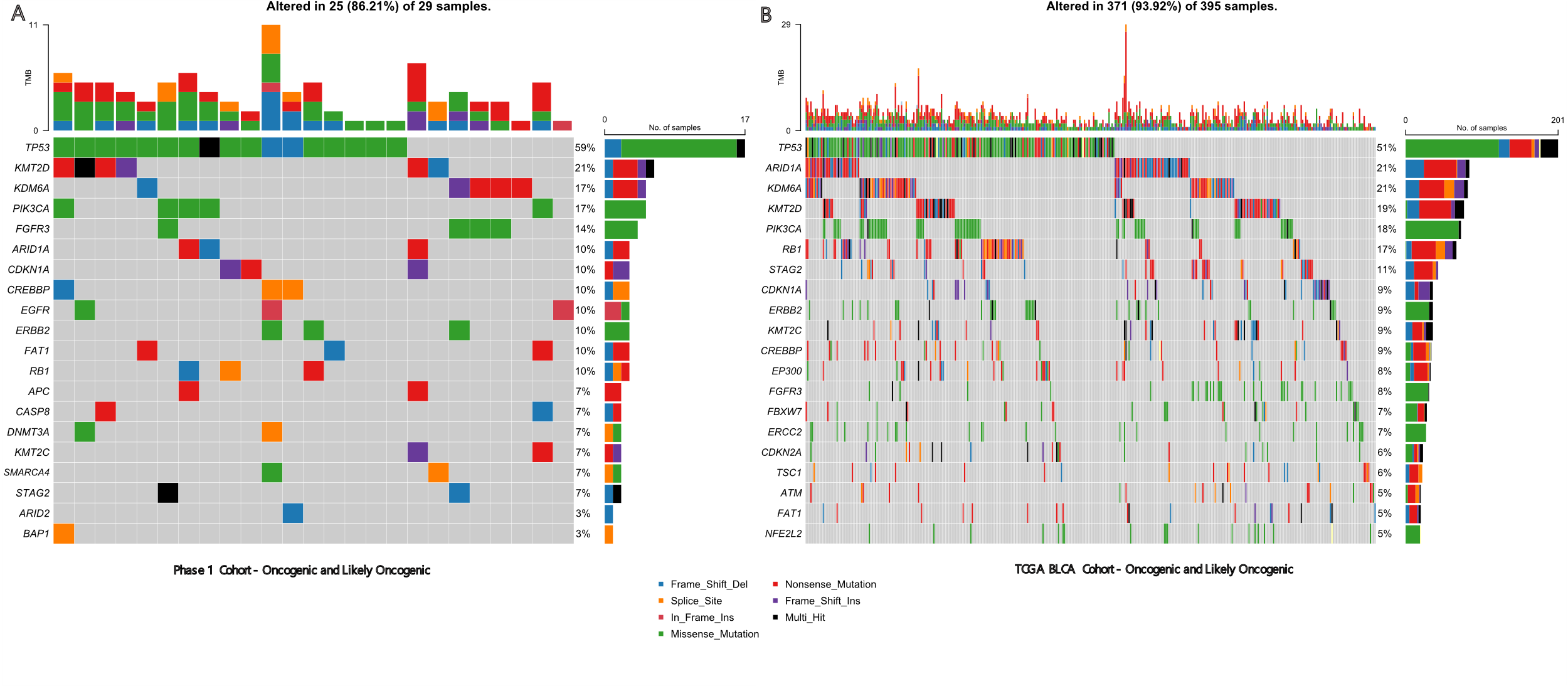


Figure S2: Top 20 oncogenic or likely oncogenic mutations in A: Phase 1 cohort. B: TCGA-cohort.


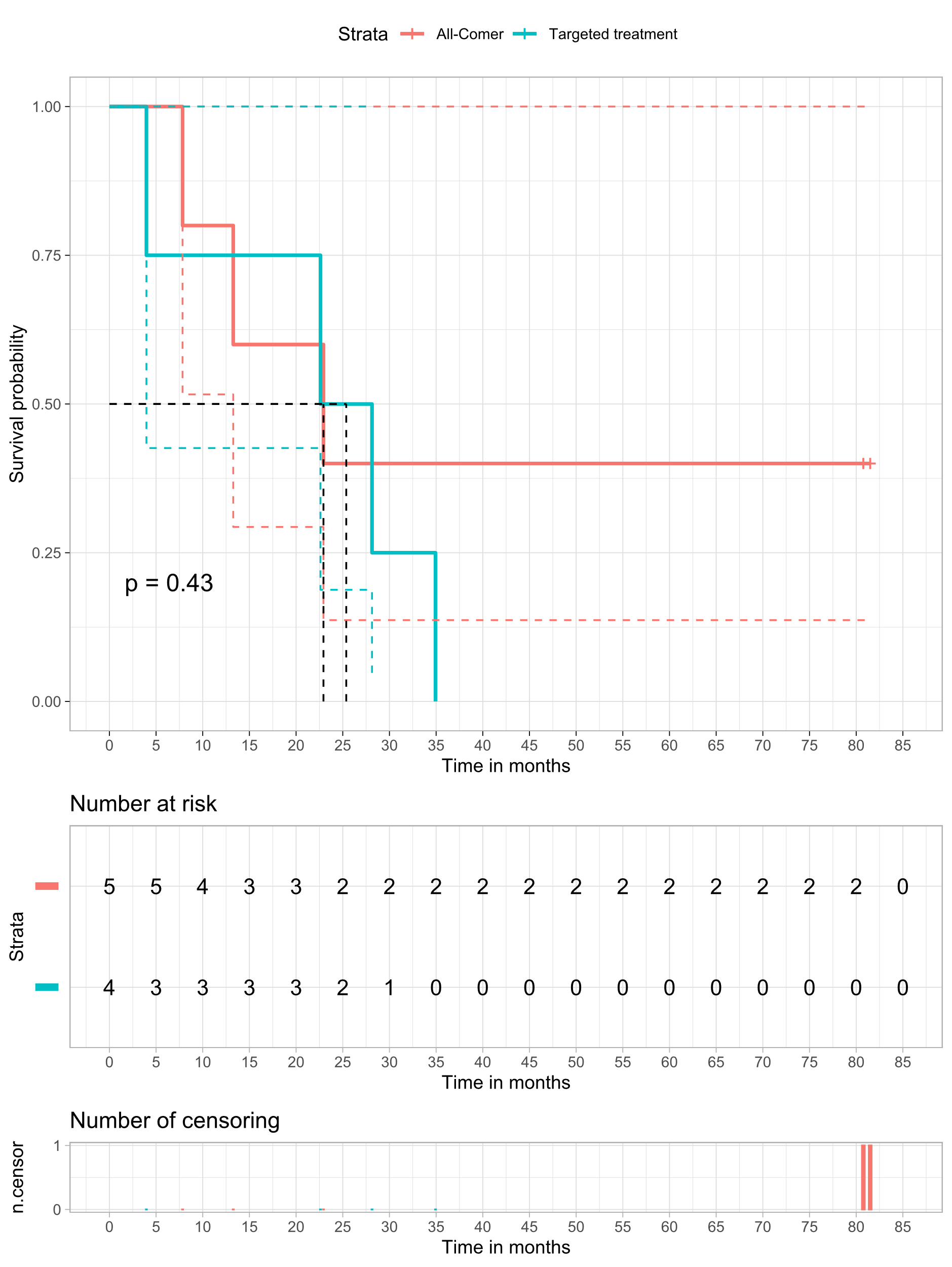


Figure S4: Survival from time of inclusion in phase 1 unit, of the nine patients included in phase 1 trial stratified by all-comer or targeted treatment (se Table S4 for specification).

| Signal Transduction and Kinase Activity | - FGFR3, FGFR2 (Fibroblast Growth Factor Receptors) |
| --- | --- |
|  | - HRAS, KRAS, NRAS (RAS family of GTPases) |
|  | - BRAF (part of the RAF family, involved in MAPK/ERK pathway) |
|  | - EGFR, ERBB2 (Epidermal Growth Factor Receptors) |
|  | - PIK3CA (Phosphoinositide 3-kinase) |
|  | - RET (Receptor Tyrosine Kinase) |
|  | - AKT1 (Serine/Threonine Kinase) |
|  | - MTOR (Mechanistic Target Of Rapamycin Kinase) |
|  | - NF1 (Neurofibromin, RAS GTPase activating protein) |
| DNA Damage Response and Repair | - TP53 (Tumor Protein p53, DNA damage response) |
|  | - ERCC2 (Nucleotide excision repair) |
|  | - BRCA1, BRCA2 (Homologous recombination repair) |
|  | - ATM (ATM Serine/Threonine Kinase, response to DNA double-strand breaks) |
|  | - ATR (ATR Serine/Threonine Kinase, DNA damage checkpoint) |
|  | - CHEK2 (Checkpoint kinase, DNA damage response) |
|  | - PALB2 (Partner and localizer of BRCA2) |
|  | - RAD51C (Homologous recombination) |
|  | - BRIP1 (BRCA1 Interacting Protein) |
|  | - FANCA (Fanconi anemia pathway) |
|  | - NBN (Nibrin, DNA double-strand break repair) |
| Chromatin Modification and Gene Expression Regulation | - ARID1A (Chromatin remodeling) |
|  | - KDM6A (Histone demethylase) |
| Cell Cycle Regulation | - CDKN2A (Cyclin-Dependent Kinase Inhibitor) |
|  | - CDK12 (Cyclin-Dependent Kinase) |
| Metabolic Pathways | - IDH1 (Isocitrate Dehydrogenase, involved in metabolism) |
| Tumor Growth and Angiogenesis | - TSC1, TSC2 (Tuberous Sclerosis Complex, regulators of mTOR pathway) |
|  | - PTEN (Phosphatase and Tensin Homolog, PI3K/AKT pathway regulator) |

Table S1: Showing areas of genetic mutations involved in cancer evolution with targetable lesions.

| First line | Second line | Third Line | CoPPO treatment |
| --- | --- | --- | --- |
| cis/gem | Vinflunin | - 🡪 | Non-targeted treatment |
| cis/gem | Vinflunin | - 🡪 | Non-targeted treatment |
| cis/gem | Vinflunin | - 🡪 | Non-targeted treatment |
| cis/gem | Vinflunin | - 🡪 | FGFR2 targeted treatment |
| cis/gem | Pembrolizumab | - 🡪 | ERBB2 targeted treatment |
| cis/gem | cis/gem reind | Vinflunin | Non-targeted treatment |
| carbo/gem | Pembrolizumab | - 🡪 | Non-targeted treatment |
| cis/gem | Pembrolizumab | Vinflunin | ERBB2 targeted treatment |
| cis/gem | cis/gem reind | Pembrolizumab | FGFR2 targeted treatment |

Table S2: Previous treatment lines for all 9 patients receiving CoPPO protocolled treatment. Arrow indicates that third line treatment was a CoPPO protocol.

| **Classifier** | **Subtype** | **Count**  **TCGA** | **Count**  **Phase1** | **Percentage**  **TCGA** | **Percentage**  **Phase1** | **p** | **p.adj** | **p.adj.signif** |
| --- | --- | --- | --- | --- | --- | --- | --- | --- |
| CIT | MC1 | 170 | 4 | 39.4 | 21.1 | 0.24 | 0.83 | ns |
| CIT | MC7 | 134 | 4 | 31.1 | 21.1 | 0.66 | 1.00 | ns |
| CIT | MC4 | 104 | 4 | 24.1 | 21.1 | 1.00 | 1.00 | ns |
| CIT | MC3 | 17 | 4 | 3.9 | 21.1 | 0.01 | 0.07 | ns |
| **CIT** | **MC6** | **3** | **3** | **0.7** | **15.8** | **0.00** | **0.01** | ***** |
| CIT | MC2 | 3 | 0 | 0.7 | 0.0 | 1.00 | 1.00 | ns |
| Lund | UroA-Prog | 102 | 4 | 23.7 | 21.1 | 1.00 | 1.00 | ns |
| Lund | Sc/NE-like | 15 | 4 | 3.5 | 21.1 | 0.01 | 0.06 | ns |
| Lund | Mes-like | 44 | 3 | 10.2 | 15.8 | 0.45 | 1.00 | ns |
| Lund | Ba/Sq | 61 | 2 | 14.2 | 10.5 | 1.00 | 1.00 | ns |
| Lund | UroC | 52 | 2 | 12.1 | 10.5 | 1.00 | 1.00 | ns |
| Lund | GU | 30 | 2 | 7.0 | 10.5 | 0.39 | 1.00 | ns |
| Lund | Ba/Sq-Inf | 42 | 0 | 9.7 | 0.0 | 0.26 | 0.83 | ns |
| Lund | Uro-Inf | 36 | 1 | 8.4 | 5.3 | 1.00 | 1.00 | ns |
| Lund | GU-Inf | 28 | 0 | 6.5 | 0.0 | 0.63 | 1.00 | ns |
| Lund | UroB | 21 | 1 | 4.9 | 5.3 | 0.61 | 1.00 | ns |
| MDA | p53-like | 122 | 9 | 28.3 | 47.4 | 0.12 | 0.55 | ns |
| MDA | luminal | 166 | 5 | 38.5 | 26.3 | 0.55 | 1.00 | ns |
| MDA | basal | 143 | 5 | 33.2 | 26.3 | 0.83 | 1.00 | ns |
| **TCGA** | **Neuronal** | **19** | **6** | **4.4** | **31.6** | **0.0001** | **0.01** | ****** |
| TCGA | Basal_squamous | 136 | 4 | 31.6 | 21.1 | 0.52 | 1.00 | ns |
| TCGA | Luminal_papillary | 136 | 4 | 31.6 | 21.1 | 0.52 | 1.00 | ns |
| TCGA | Luminal_infiltrated | 96 | 3 | 22.3 | 15.8 | 0.80 | 1.00 | ns |
| TCGA | Luminal | 44 | 2 | 10.2 | 10.5 | 1.00 | 1.00 | ns |
| UNC | Basal | 214 | 11 | 49.7 | 57.9 | 0.60 | 1.00 | ns |
| UNC | Luminal | 217 | 8 | 50.3 | 42.1 | 0.73 | 1.00 | ns |
| Concensus | Ba/Sq | 153 | 3 | 35.5 | 15.8 | 0.21 | 0.83 | ns |
| Concensus | LumP | 147 | 4 | 34.1 | 21.1 | 0.41 | 1.00 | ns |
| Concensus | LumU | 52 | 5 | 12.1 | 26.3 | 0.09 | 0.48 | ns |
| **Concensus** | **NE-like** | **6** | **4** | **1.4** | **21.1** | **0.00** | **0.01** | ****** |
| Concensus | Stroma-rich | 54 | 3 | 12.5 | 15.8 | 0.73 | 1.00 | ns |
| Concensus | LumNS | 19 | 0 | 4.4 | 0.0 | 1.00 | 1.00 | ns |

Table S3: Molecular subtype by RNA-expression according to the classifiers as described in [1]. Bold indicates significant difference.

1. Robertson, A.G.; Kim, J.; Al-Ahmadie, H.; Bellmunt, J.; Guo, G.; Cherniack, A.D.; Hinoue, T.; Laird, P.W.; Hoadley, K.A.; Akbani, R.; et al. Comprehensive Molecular Characterization of Muscle-Invasive Bladder Cancer. *Cell* **2017**, *171*, 540-556.e25, doi:10.1016/J.CELL.2017.09.007.

| Kinase Inhibitors | Erdafitinib: FGFR3, FGFR2 |
| --- | --- |
|  | Tipifarnib: HRAS |
|  | Trametinib: BRAF, KRAS, NF1 |
|  | PLX8394: BRAF |
|  | Amivantamab: EGFR |
|  | Poziotinib: EGFR |
|  | CLN-081: EGFR |
|  | Mobocertinib: EGFR |
|  | Neratinib: ERBB2 |
|  | Selpercatinib: RET |
|  | Alpelisib: PIK3CA |
|  | AZD5363: AKT1 |
|  | RLY-2608: PIK3CA |
|  | Binimetinib: NRAS, KRAS |
|  | Cobimetinib: KRAS, NF1 |
|  | RMC-6236: KRAS |
|  | MRTX-1133: KRAS |
|  | RLY-4008: FGFR2 |
|  | AZD4547: FGFR2 |
|  | GSK2636771: PTEN |
|  | AZD8186: PTEN |
|  | Sotorasib: KRAS |
| DNA Damage Response Modulators | Cisplatin: ERCC2 |
|  | Olaparib: BRCA1, BRCA2, ATM, ATR, BRIP1, CDK12, CHEK2, PALB2, RAD51C |
|  | Talazoparib: BRCA1, FANCA, NBN, ATM, ATR, CDK12, CHEK2, RAD51C |
|  | Rucaparib: BRCA1, BRCA2, PALB2 |
|  | Niraparib: BRCA1, BRCA2 |
|  | Tazemetostat: ARID1A, KDM6A |
|  | PLX2853: ARID1A |
| Monoclonal Antibodies and Antibody-Drug Conjugates | Ado-Trastuzumab Emtansine: ERBB2 |
|  | Trastuzumab Deruxtecan: ERBB2 |
|  | Trastuzumab+Pertuzumab+Docetaxel: ERBB2 |
| mTOR Inhibitors | Everolimus: TSC1, TSC2, MTOR |
|  | Temsirolimus: MTOR |
|  | ABI-009: TSC2 |
| CDK Inhibitors | Palbociclib: CDKN2A |
|  | Ribociclib: CDKN2A |
|  | Abemaciclib: CDKN2A |
| IDH Inhibitors | Ivosidenib: IDH1 |
|  | Vorasidenib: IDH1 |
| Other Combinations | Olaparib+Bevacizumab: BRCA1, BRCA2 |
|  | Talazoparib+Enzalutamide: FANCA, NBN |
|  | Alpelisib+Fulvestrant: PIK3CA |
|  | Adagrasib+Cetuximab: KRAS |
|  | Adagrasib+Panitumumab: KRAS |
|  | Sotorasib+Cetuximab: KRAS |
|  | Sotorasib+Panitumumab: KRAS |
|  | ASP3082: KRAS |
|  | PC14586: TP53 |

Table S4: Treatments targeting specific mutations found in bladder cancer cohorts (TCGA and Phase 1 cohort). All references and matching variants are shown in Appendix 2.
